# Torsion of a necrotic appendiceal mucocele presenting as acute abdomen: a rare case report

**DOI:** 10.1093/jscr/rjag409

**Published:** 2026-05-31

**Authors:** Efstratios Kouskos, Panagiota Dalla, Panagiotis Eirineos Kouskos, Apostolos Andronikou

**Affiliations:** Surgical Department, General Hospital “Vostanio”, Mytilene 81100, Lesvos Island, Greece; Surgical Department, General Hospital “Vostanio”, Mytilene 81100, Lesvos Island, Greece; School of Medicine, European University Cyprus, Egkomi, 2404 Nicosia, Cyprus; Surgical Department, General Hospital “Vostanio”, Mytilene 81100, Lesvos Island, Greece

**Keywords:** appendiceal mucocele, appendiceal torsion, acute abdomen, pseudomyxoma peritonei, emergency surgery

## Abstract

Torsion of the vermiform appendix is rare. When torsion involves an appendiceal mucocele, timely surgical management, and avoidance of rupture are essential to prevent intraperitoneal mucin spillage. A 68-year-old woman presented with 12 h history of severe lower abdominal pain. Examination revealed marked lower abdomen rebound tenderness and her laboratory tests showed leukocytosis and elevated C-reactive protein. Urgent computed tomography demonstrated a 9 × 3.4 cm predominantly cystic mass posterior to the uterus with mural calcifications, consistent with an appendiceal mucocele. Emergency laparotomy revealed a necrotic appendiceal mucocele with complete torsion. The lesion was resected intact. Histopathology confirmed an appendiceal mucocele. The patient had an uncomplicated recovery and remains in excellent condition 3 months postoperatively. Appendiceal mucocele torsion should be considered in acute abdomen with a cystic pelvic mass on computed tomography. Intact resection is a key operative principle.

## Introduction

Appendiceal mucoceles are uncommon lesions (0.2%–0.3% of appendectomy specimens) characterized by dilatation of the appendix due to mucin accumulation. Their formation is usually secondary to mucinous appendiceal neoplasms which are classified as mucosal adenoma, low-grade appendiceal mucinous neoplasm (LAMN), high grade mucinous adenocarcinoma, and signet-ring cell carcinoma. On the other hand there are non-neoplastic variants as mucosal hyperplasia and mucosal retention cyst [1]. Clinical presentation ranges from incidental findings to acute abdomen.

Torsion of the appendix is rare and may occur spontaneously, without any primary lesion, or secondary to an underlying appendiceal lesion, including fecalith, cystadenoma, carcinoid tumor, adhesion, lipoma, or mucocele. Factors affecting primary torsion include long appendix, narrow base, and a fan-shaped mesoappendix. When torsion develops, vascular compromise can lead to gangrene and peritonitis [2]. Additionally, rupture of a mucocele can be associated with intraperitoneal dissemination of mucin related to a very severe complication of PseudoMixoma Peritonei (PMP), underlining the importance of careful operative handling and intact specimen extraction [3].

We report a case of complete torsion and necrosis of an appendiceal mucocele presenting as acute abdomen with a pelvic-appearing mass on computed tomography (CT) and managed with emergency open resection.

## Case report

A 68-year-old woman presented to the emergency department with 12 h of severe lower abdominal pain. On clinical examination, she had marked rebound tenderness in the right and midline lower abdomen. Her medical history was negative for comorbidities and prior operations.

Laboratory investigations showed a white blood cell count of 11 800/μL and C-reactive protein of 125 mg/L.

**Figure 1:**
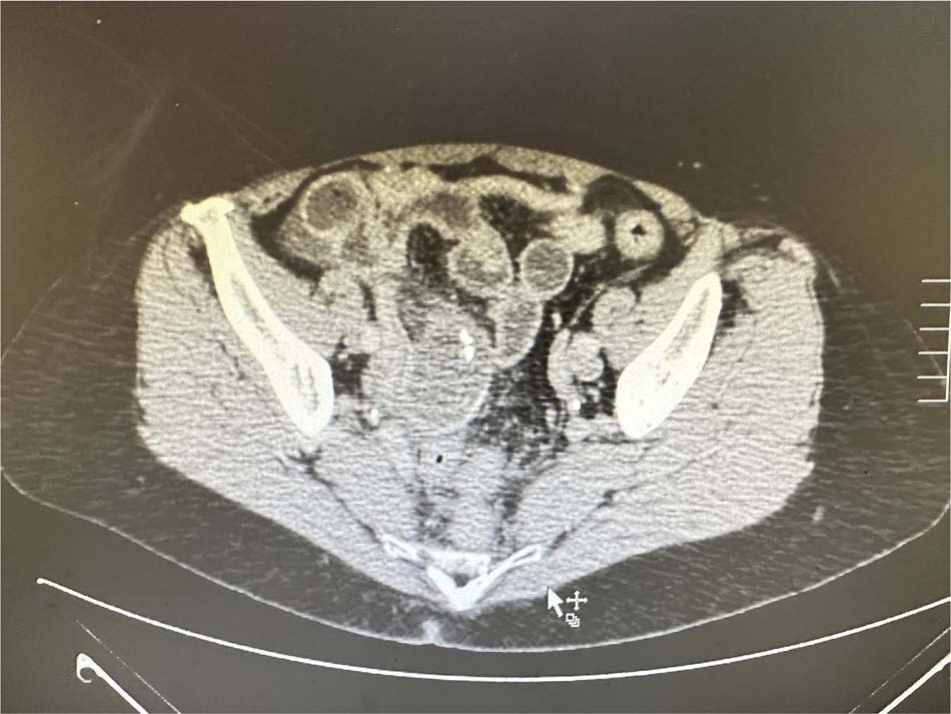
CT revealed a large predominantly cystic mass posterior to the uterus with mural calcifications and apparent continuity with the appendix, indicating an appendiceal mucocele.

Urgent CT revealed a 9 × 3.4 cm predominantly cystic mass posterior to the uterus with mural calcifications and apparent continuity with the appendix, suggestive of appendiceal mucocele (Fig. 1).

**Figure 2:**
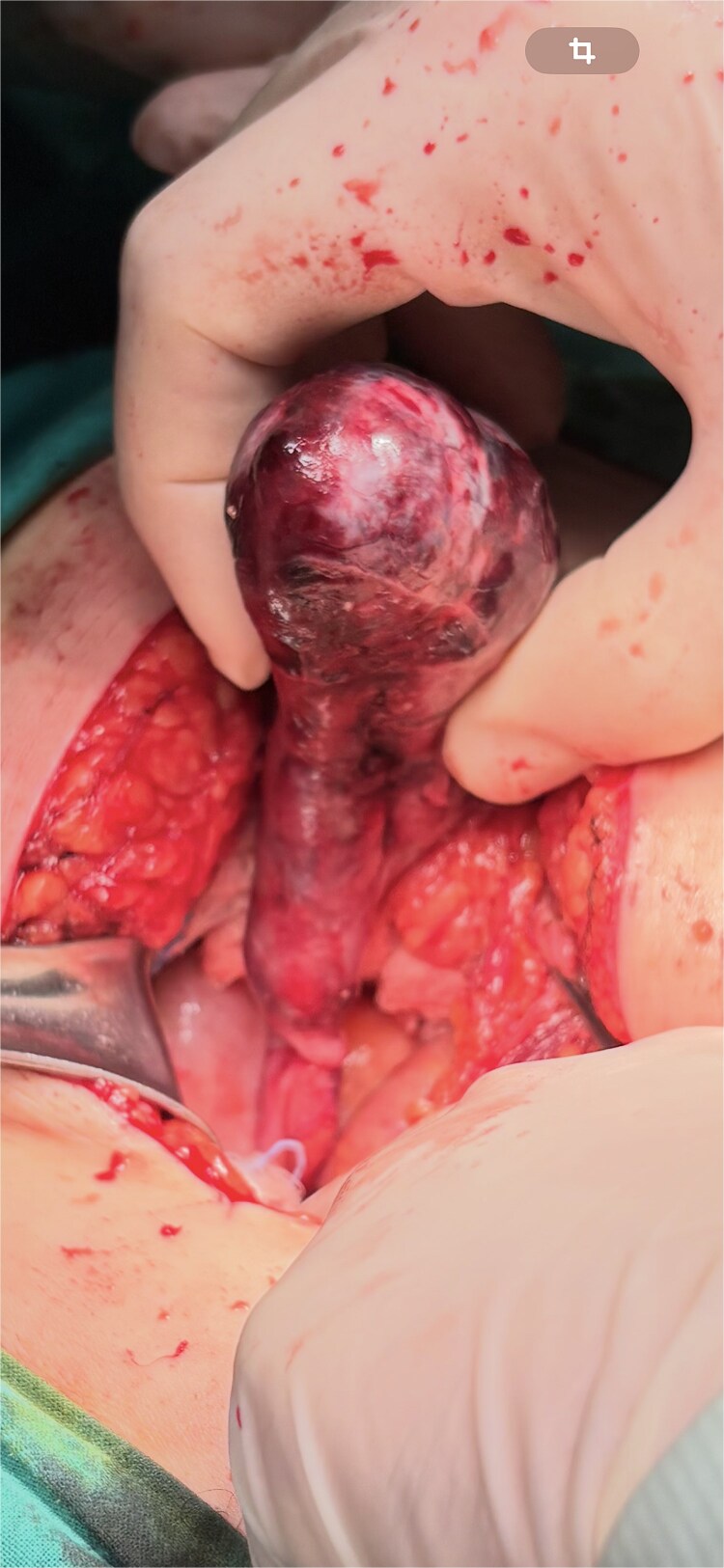
Ischemic appendiceal mucocele with complete torsion (˃360^°^).

**Figure 3:**
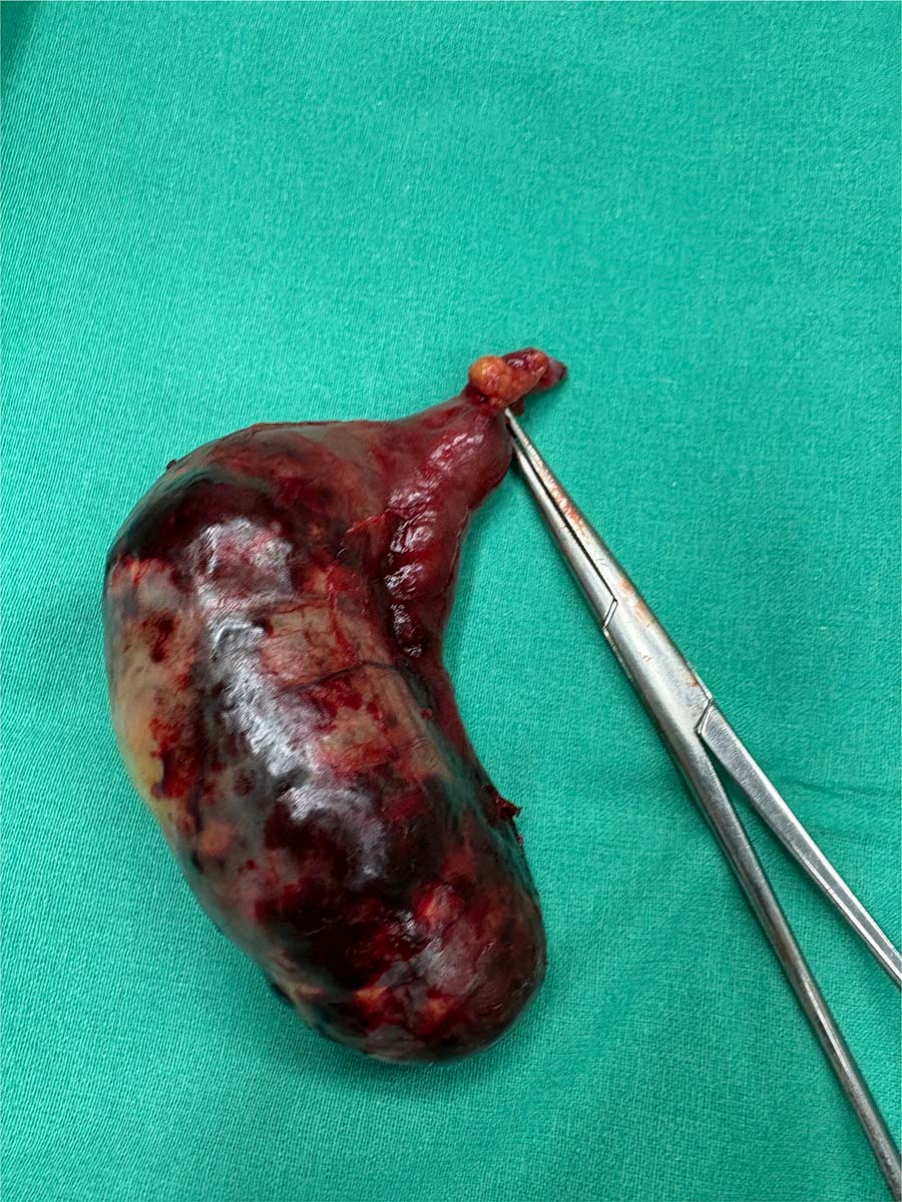
Surgical specimen: necrotic appendiceal mucocele, removed intact, and trancected at a macroscopically normal appendiceal margin.

Given peritonism and radiologic concern for complicated appendiceal pathology, the patient underwent emergency surgery through a midline supraumbilical incision. Intraoperatively, a necrotic appendiceal mucocele with complete torsion (˃360^°^) was identified (Fig. 2). The lesion was removed intact. Transection was performed at a macroscopically normal appendiceal tissue proximal to the torsion site, with a grossly clear margin (Fig. 3).

Histopathological examination confirmed a simple appendiceal mucocele. No signs of malignancy on mucocele wall were found and the content was mucin with no epithelial cells. The postoperative course was uncomplicated. The patient was discharged on postoperative day 2 in excellent condition and remains so, clinically and by CT plus colonoscopy, 3 months later.

## Discussion

Torsion of an appendiceal mucocele is a rare cause of acute abdomen. Preoperative diagnosis can be challenging because symptoms and laboratory abnormalities overlap with complicated acute or perforated appendicitis and gynecologic pathology (e.g. adnexal torsion or complex ovarian cyst).

CT is the most helpful diagnostic tool and can suggest mucocele when there is a cystic/tubular mass related to the appendix, sometimes with wall calcifications. It may raise suspicion for torsion when associated with features of ischemia or abnormal orientation. Case literature describing torsed mucoceles supports the role of cross-sectional imaging in preoperative suspicion, even though definitive diagnosis is commonly intraoperative [4].

In our patient, CT suggested a cystic appendiceal lesion with calcified wall elements, prompting urgent operative management.

From a surgical perspective, the key principle is careful handling and intact resection to avoid rupture and mucin spillage. Multiple sources stress that rupture of an appendiceal mucocele—spontaneous or iatrogenic—can seed mucin into the peritoneum and contribute to PMP, particularly when neoplastic mucinous pathology exists. Consequently, many authors advocate careful handling and intact removal of the lesion [5, 6].

An open approach can be advantageous in selected cases to provide direct control, facilitate gentle handling, and allow safe specimen extraction—particularly for larger lesions or when anatomy is distorted. Laparoscopic management has been reported (including series describing totally laparoscopic resection without rupture). However, approach selection should be individualized based on surgeon expertise, lesion size, and intraoperative conditions [3, 7].

Treatment of appendicular torsion depends on the cause. Appenticectomy is sufficient in case of primary torsion without abnormal lesions, mucosal hyperplasia, mucosal retention cyst, mucosal adenoma, and LAMN limited to appendix. In case of cystadenocarcinoma, a right hemicolectomy with regional lymphadenectomy is necessary. The same operation is required in case of a mucinous cystadenocarcinoma diagnosis in histopathological examination of appendicectomy specimen [5].

In conclusion, the combination of acute peritonism, inflammatory markers, and CT findings highly indicate torsion of an appendiceal mucocele and prompted emergency operative management is required.

## Data Availability

Data are available from the corresponding author on a reasonable request.
